# Reduced Cortical Complexity in Cirrhotic Patients with Minimal Hepatic Encephalopathy

**DOI:** 10.1155/2020/7364649

**Published:** 2020-03-18

**Authors:** Qiu-Feng Chen, Xiao-Hong Zhang, Tian-Xiu Zou, Nao-Xin Huang, Hua-Jun Chen

**Affiliations:** ^1^College of Computer and Information Sciences, Fujian Agriculture and Forestry University, Fuzhou 350002, China; ^2^Department of Radiology, Fujian Medical University Union Hospital, Fuzhou 350001, China; ^3^Department of Radiology, The First Affiliated Hospital of Nanjing Medical University, Nanjing 210029, China

## Abstract

**Purpose:**

Gray matter volume loss, regional cortical thinning, and local gyrification index alteration have been documented in minimal hepatic encephalopathy (MHE). Fractal dimension (FD), another morphological parameter, has been widely used to describe structural complexity alterations in neurological or psychiatric disease. Here, we conducted the first study to investigate FD alterations in MHE.

**Methods and Materials:**

We performed high-resolution structural magnetic resonance imaging on cirrhotic patients with MHE (*n* = 20) and healthy controls (*n* = 21). We evaluated their cognitive performance using the psychometric hepatic encephalopathy score (PHES). The regional FD value was calculated by Computational Anatomy Toolbox (CAT12) and compared between groups. We further estimated the association between patients' cognitive performance and FD values.

**Results:**

MHE patients presented significantly decreased FD values in the left precuneus, left supramarginal gyrus, right caudal anterior cingulate cortex, right isthmus cingulate cortex, right insula, bilateral pericalcarine cortex, and bilateral paracentral cortex compared to normal controls. In addition, the FD values in the right isthmus cingulate cortex and right insula were shown to be positively correlated with patients' cognitive performance.

**Conclusion:**

Aberrant cortical complexity is an additional characteristic of MHE, and FD analysis may provide novel insight into the neurobiological basis of cognitive dysfunction in MHE.

## 1. Introduction

As a frequent neurocognitive complication of cirrhosis, hepatic encephalopathy (HE) results from the escape of substances from the liver, which can cause hyperammonemia or other metabolic disturbance [1]. It can result in a wide variety of symptoms, from slight cognitive impairment up to coma or even death. The mildest form of HE is minimal hepatic encephalopathy (MHE), which usually lacks obvious symptoms and can only be diagnosed through neurophysiological or neuropsychological testing [1, 2]. However, MHE negatively impacts quality of life [3] and can increase the risk of traffic accidents [4] and predict poor prognosis [5].

High-resolution magnetic resonance imaging (MRI) has helped to advance our understanding of the mechanisms and etiology of MHE. MHE patients have diffuse cerebral cortical structure abnormalities. For example, the voxel-based morphometry (VBM) studies have indicated that cirrhotic patients with MHE have reduced gray matter volume, primarily in the middle and inferior frontal cortex, anterior cingulate cortex, putamen, paracentral lobule, and cerebellum posterior lobe [6–8]. Similarly, focusing on brain tissue density, another study found brain tissue concentration reductions predominantly in the precuneus, paracentral lobule, putamen, and middle frontal cortex of cirrhotic patients [9]. Furthermore, the extents and sizes of abnormal brain areas run parallel with the degree of liver failure and patients' neurocognitive dysfunction [9, 10]. In addition, structural magnetic resonance imaging using surface-based morphometry (SBM) has found an abnormally decreased thickness of the superior temporal cortex and precuneus cortex in MHE patients relative to healthy controls [11]. Moreover, cognitive deficits have been associated with brain surface structural changes, indicating that these alterations affect patient neurocognitive abilities. A vertex-based shape analysis has also suggested that cirrhosis is associated with nonuniform distributed shape abnormalities in the deep gray matter and more regions of the deep gray matter are affected as the disease progresses [12].

Given that different measures may index distinct characteristics of cortical structure [13], it is probable that establishing other cortical measurements in MHE and comparing these profiles to those of well-matched healthy controls may provide additional information regarding the underlying anatomical correlates of MHE. Fractal dimension (FD) analysis was originally developed for the use of fractals (structures that are self-similar in a scale-free manner [14]) but has also been used to quantify brain structural complexity [15, 16], which condenses the cortical thickness, sulcal depth, and folding area and generates unique numerical values [17]. Using appropriately scaled and increasingly smaller sampling or measuring instruments, FD has been used to quantify the statistical properties of the cerebral cortex [18]. As a complement to other structural measurements [19, 20], FD quantitatively measures the complexity of the brain and shows significant correlation with other index such as cortical thickness, curvature, and gyrification index [13, 21]. In addition, a number of studies have elucidated the correlation between cortical complexity and neurocognitive performance [13, 19]. FD measures have successfully revealed structural complexity changes in aging [13, 22], schizophrenia [18], Williams syndrome [23], Alzheimer's disease [19, 21], and other neuropsychiatric disorders [24, 25], and they show greater sensitivity in detecting cerebral structural changes than other measurements, to some extent [13, 26].

However, there are still no studies regarding the FD differences between MHE and healthy controls. Thus, for the first time, we investigated the structural complexity (indexed by FD) alterations in MHE and the correlation between regional FD and patient cognitive performance (as measured by psychometric hepatic encephalopathy score (PHES)).

## 2. Materials and Methods

### 2.1. Subjects

We enrolled cirrhotic patients with MHE (*n* = 20) and healthy controls (*n* = 21). Clinical and demographic characteristics are summarized in Table 1. Between the two groups, there were no significant differences in age, education level, or gender. We performed PHES examinations that included the digital symbol test (DST), serial dotting test (SDT), number connection test A (NCTA), number connection test B (NCTB), and the line tracing test (LTT) to diagnose MHE, as previously reported [27]. We excluded subjects who (1) currently had a diagnosis of overt HE or other neuropsychiatric disorder, (2) were currently taking psychotropic medications, (3) had been diagnosed with uncontrolled metabolic or endocrine diseases (e.g., thyroid dysfunction), (4) had a history of alcohol abuse within six months before the study, or (5) had other contraindications that prevented MRI. This study was approved by the Research Ethics Committee of Fujian Medical University Union Hospital and The First Affiliated Hospital of Nanjing Medical University, China. Written informed consent was obtained from all participants prior to the study.

### 2.2. MRI Data Acquisition

We used a 3.0 T scanner (Siemens, Verio, Germany) to perform MRI. We used the following parameters to collect three-dimensional high-resolution T1-weighted magnetization prepared rapid gradient echo (MPRAGE) sagittal images: TR = 1.9 ms, TE = 2.48 ms, matrix = 256 × 256, FOV = 256 × 256 mm, flip angle = 9°, slice thickness = 1.0 mm (interslice gap = 0 mm), and 176 slices.

### 2.3. Calculation of Fractal Dimension

High-resolution T1-weighted images were preprocessed, and the cortical FD was estimated using the standard protocol of the Computational Anatomy Toolbox (CAT12, http://dbm.neuro.uni-jena.de/cat/) in Statistical Parametric Mapping software (SPM12, https://www.fil.ion.ucl.ac.uk/spm/). Default settings were used for all procedures (http://www.neuro.uni-jena.de/cat12/CAT12-Manual.pdf). Image preprocessing included correction for bias-field inhomogeneities; segmentation into grey matter, white matter, and cerebrospinal fluid; and normalization (into Montreal Neurological Institute (MNI) space) using DARTEL algorithm (Diffeomorphic Anatomic Registration Trough Exponentiated Lie algebra [28]).

Then, FD was estimated following the workflow specified by Yotter et al. [14] as implemented in CAT12. This workflow allows for cortical thickness measurement and central surface reconstructions to be completed in one, fully automated step [29]. Then, a spherical harmonic method [30] was employed to reparameterize the cortical surface mesh based on an algorithm that reduces area distortions [31], in order to repair the topological defects. Finally, the approach of “spherical harmonic reconstructions” proposed by Yotter et al. [14] was used to measure the local fractal dimension, which quantifies the cortical surface complexity.

Mean FD values were computed for 34 ROIs (regions of interest) defined by the Desikan-Killiany Atlas [32] using standard procedures for “ROI analysis” provided in the CAT12 toolbox. The atlas (defined in template space) was transformed to native subject space using the inverse nonlinear deformations needed to spatially normalize images to template space. The regional FD estimation was performed in the native space (see http://dbm.neuro.uni-jena.de/cat12/CAT12-Manual.pdf). The estimated FD values in ROIs were compared across the two groups. We set the statistical threshold at a false discovery rate- (FDR-) corrected value of *P* < 0.05.

### 2.4. Correlation Analysis

The mean FD values in the ROIs that survived during between-group comparisons were extracted. The relationship between FD values in the ROIs and cognition assessments in the patients was subjected to Spearman correlation analysis. The FDR-corrected *P* value < 0.05 was treated at statistically significant.

## 3. Results

MHE patients performed worse on PHES tests compared to healthy controls (as indicated by longer times to complete SDT, NCTA, and NCTB tests and lower DST and LTT scores), suggesting cognitive impairment.

**Figure 1:**
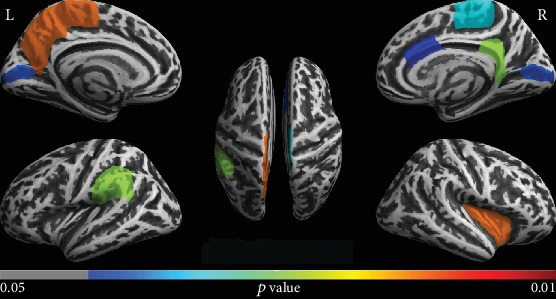
Decreased fractal dimension (FD) regions in MHE patients compared to healthy controls. These results were corrected for multiple comparisons (*P* < 0.05, FDR correction). The color bar indicates the corrected *P* value.

The ROI-based analysis showed decreased FD in MHE patients compared to healthy controls in the left precuneus, left paracentral gyrus, left supramarginal gyrus, and left pericalcarine cortex and in the right caudal anterior cingulate cortex, right pericalcarine cortex, right isthmus cingulate cortex, right insula cortex, and right paracentral cortex. No regions showed decreased FD for the healthy controls in comparison to MHE patients. Results of significant group differences are displayed in Figure 1.

**Figure 2:**
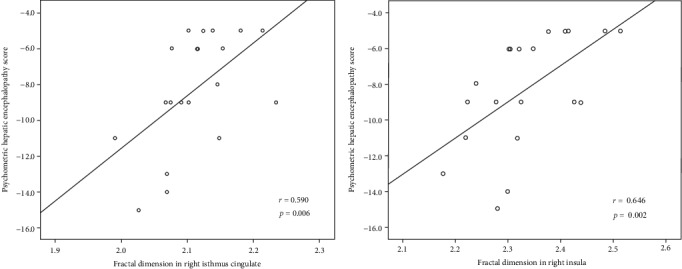
Positive correlations between FD of the right isthmus cingulate and right insula and PHES among MHE patients.

We also investigated whether cognitive performance (PHES) was associated with FD values in the ROIs in Figure 1. As shown in Figure 2, there was a significantly positive correlation between patients' cognitive performance and FD values of the right isthmus cingulate and right insula.

## 4. Discussion

We investigated cerebral cortical structural complexity using FD in MHE patients and healthy controls. We revealed significant FD reductions among MHE patients in several regions, including DMN-related (default mode network) regions (i.e., left precuneus, left supramarginal gyrus, and right isthmus cingulate cortex), visual-related regions (bilateral pericalcarine cortex), motor-associated regions (bilateral paracentral gyrus), the right insula, and the right caudal anterior cingulate cortex. Within the MHE group, we also found a significantly positive correlation between neurocognitive performance and FD of the right isthmus cingulate and right insula, suggesting that FD could be an alternative index to indicate the neurophysiological characteristics of MHE.

It has been suggested that FD reductions may result from brain inflammatory-related processes (e.g., inflammatory infiltration, loss of cell shape, necrosis, or irregular tissue organization) [33]. Previous studies have persistently reported the activation of microglia cells, increased inflammatory factor levels (e.g., TNF, IL-6, and IL-1*β*) [34, 35], Alzheimer's type II astrocytosis [36, 37], and neuronal cell death [38] in patients with liver failure. Therefore, it is expected that cirrhotic patients with MHE showed reduced fractal dimension in multiple brain regions. In fact, the reduction of cortical complexity in the microstructure level has been revealed by the diffusion imaging method (i.e., diffusion kurtosis imaging) in cirrhotic patients, which involve the diffuse areas (such as DMN- and motor-related regions) and is associated with patients' cognitive impairments [39]. Thus, our results yielded the paralleling evidence, in the relatively macroscopic level, about MHE-induced alteration in cortical structural complexity, in addition to previous report. Furthermore, we found that these regions with a decreased FD value in MHE patients also showed reduced gray matter density that was revealed by a previous VBM study [9]. This consistence in spatial distribution may further suggest that these cortical areas are the crucial nodes affected by MHE-related pathological processes.

Cirrhotic patients with MHE had decreased FD in DMN-related regions (i.e., left precuneus, right isthmus cingulate cortex, and left supramarginal gyrus) compared to healthy controls. Many studies have consistently reported that the structural alterations in the precuneus and posterior cingulate cortex, such as regional atrophy, cortical thinning, and decreased microstructural complexity, are the important characteristics occurring in cirrhotic patients, which parallels with cognitive dysfunctions [9, 11, 39]. Also, the supramarginal gyrus has been found to show abnormal microstructure in cirrhosis [39]. What is more, it is noted that these three regions represent the important constituents of DMN. Both structural and functional MRIs have consistently demonstrated that MHE is characterized by abnormalities in the DMN, which is responsible for the cognitive deficits in MHE patients. For instance, structural MRI has demonstrated reduced gray matter density within DMN-related regions in cirrhotic patients with MHE [9]. Consistently, spontaneous brain activity [40] and intrinsic functional connectivity [41–43] reductions within DMN-related regions have been reported and are correlated with the poor neurocognitive performance in MHE patients [40, 44]. The DMN is functionally characterized by being activated in the resting state and deactivated when performing attention-demanding tasks, which is essential in reallocating neuronal resources toward those processes [45]. It has been reported that MHE patients present impaired selective attention and sustained attention [46]. Thus, we infer that the impaired cortical complexity in these regions may relate to the attention deficits in MHE.

It has been reported that MHE patients have reduced visual abilities such as impaired visual judgment, visual-spatial reasoning, and visual-motor coordination. As expected, in this study, MHE patients showed decreased cortical complexity in visual-related regions (pericalcarine cortex). Consistently, the altered cortical morphometry in the pericalcarine region has been found in the cirrhotic patients and is correlated with cognitive deficits [47]. In fact, reduced synchronicity of neural activity and functional connectivity within visual-relevant regions have been reported in MHE and are correlated with patients' poor neurocognitive performance [48]. Hence, the decreased cortical complexity in the visual cortex may also account for such visual deficits in MHE. The paracentral cortex, a motor-related region, was found to have decreased cortical complexity in MHE. In consistent with that, the VBM study has shown the significant reduction of gray matter density in the paracentral cortex among cirrhotic patients with MHE [9]. Similarly, previous studies have demonstrated reduced neural activity in motor-related regions [49, 50]. Also, MHE patients show disturbed resting-state functional connectivity in the paracentral cortex [27]. Of potential functional relevance, the paracentral cortex is associated with extremity movement and attention to somatosensory stimulation [51, 52]. Thus, we can infer that dysfunction of the paracentral cortex might constitute an important neural source of the impaired motor ability in MHE.

Additionally, MHE patients also show impaired cortical complexity in the insula. Consistently, the decreased insula volume and cortical complexity have been revealed in MHE [39, 44]. Moreover, it has been reported that resting-state neuronal activity shows abnormal regional homogeneity (ReHo, which reflects intrinsic brain activity local synchrony) in MHE patient insula [53, 54]. It has been proposed that the insula integrates internal and external stimuli and is of great importance in episodic memory processing [55] and in switching between two major networks (the central executive network (CEN) and the DMN) [56]. The CEN is engaged in processing attention-associated behaviors, while the DMN shows reduced neuronal activity. Therefore, abnormal cortical complexity in the insula may be the neurobiological basis of the neurocognitive deficits in MHE, which is further supported by the positive link between neurocognitive performance and FD of the right insula in MHE. The caudal anterior cingulate also showed decreased FD in MHE in comparison to healthy controls. In keeping with that, the metabolic disturbance [57], regional atrophy [58], and the damage to microstructural complexity [39] have been demonstrated in the anterior cingulate cortex among cirrhotic patients, which associate with patients' cognitive dysfunction. Since the caudal anterior cingulate is strongly implicated in cognitive information processing, including attention, salience, interference, and response competition [59, 60], our findings appear consistent with previous studies indicating that MHE is characterized by impairments in these cognitive domains.

We acknowledge that there are several limitations to this study. First, the statistical power was limited by the relatively small sample size. Additional studies with more participants will be needed to corroborate these results. Second, as this was a cross-sectional study, we did not investigate the developmental dynamics of the structural abnormalities from MHE to full HE in cirrhotic patients. More studies will be needed to observe the changing patterns in structural abnormality along with the disease progression. Third, we confined our attention to the cerebral cortex, while other studies have shown that the subcortical region is another pathological node of MHE. It will greatly facilitate our comprehensive understanding of the mechanism underlying MHE to investigate both cortical and subcortical alterations in future studies.

## 5. Conclusions

In summary, MHE patients showed a decreased cortical complexity in several regions serving cognitive function, such as attention, visual, and motor ability. The correlation between FD and PHES results suggested that cortical complexity may be a potential biomarker for neurocognitive impairments in MHE. Thus, aberrant cortical complexity is considered as an additional characteristic of MHE and FD analysis may provide novel insight into the neurobiological basis of cognitive dysfunctions in MHE.

## Figures and Tables

**Table 1:** Subject clinical and demographic information.

	HC subjects (*n* = 21)	MHE patients (*n* = 20)	*P* value
Age (year)	48.9 ± 10.9	50.0 ± 8.2	0.30
Gender (male/female)	15/6	16/4	0.78 (*χ*^2^ test)
Education level (year)	9.1 ± 3.8	8.7 ± 2.8	0.15
Etiology of cirrhosis (HBV/alcoholism/other)	—	13/4/3	—
Child-Pugh stage (A/B/C)	—	3/13/4	—
PHES tests			
Final PHES (score)	0.6 ± 1.5	−8.3 ± 3.2	<0.001
Number connection test A (seconds)	33.6 ± 8.8	58.4 ± 17.4	<0.001
Number connection test B (seconds)	53.7 ± 19.6	136.7 ± 64.7	<0.001
Serial dotting test (seconds)	39.1 ± 6.4	64.8 ± 19.2	<0.001
Digit symbol test (raw score)	50.2 ± 13.8	27.3 ± 8.8	< 0.001
Line tracing test (raw score)	110.0 ± 21.8	190.3 ± 49.3	< 0.001

Abbreviations: MHE: minimal hepatic encephalopathy; HC: healthy control; PHES: psychometric hepatic encephalopathy score; HBV: hepatitis B virus.

## Data Availability

The MRI and clinical data used to support the findings of this study are available from the corresponding author upon request.

## Abbreviations

HE: Hepatic encephalopathy
MHE: Minimal hepatic encephalopathy
FD: Fractal dimensionality
PHES: Psychometric hepatic encephalopathy score
CAT12: Computational Anatomy Toolbox
DST: Digital symbol test
NCTA: Number connection test A
NCTB: Number connection test B
SDT: Serial dotting test
LTT: Line tracing test
DMN: Default mode network
DKI: Diffusion kurtosis imaging
CEN: Central executive network.
